# All-cause mortality around the anniversary of a sibling’s death: findings from Swedish National Register Data

**DOI:** 10.1093/aje/kwaf213

**Published:** 2025-09-30

**Authors:** Sandra Rogne, Alessandra Grotta, Can Liu, Lisa Berg, Jan Saarela, Ichiro Kawachi, Ayako Hiyoshi, Mikael Rostila

**Affiliations:** Department of Public Health Sciences, Stockholm University, Stockholm, Sweden; Centre for Health Equity Studies (CHESS), Stockholm University/Karolinska Institutet, Stockholm, Sweden; Department of Public Health Sciences, Stockholm University, Stockholm, Sweden; Centre for Health Equity Studies (CHESS), Stockholm University/Karolinska Institutet, Stockholm, Sweden; Department of Medical Epidemiology and Biostatistics, Karolinska Institutet, Stockholm, Sweden; Centre for Health Equity Studies (CHESS), Stockholm University/Karolinska Institutet, Stockholm, Sweden; Clinical Epidemiology Unit, Department of Medicine, Solna, Karolinska Institutet, Stockholm, Sweden; Centre for Health Equity Studies (CHESS), Stockholm University/Karolinska Institutet, Stockholm, Sweden; Demography Unit, Faculty of Education and Welfare Studies, Åbo Akademi University, Vaasa, Finland; University of Helsinki, Helsinki, Finland; Department of Social and Behavioral Sciences, Harvard School of Public Health, Boston, MA, United States; Department of Public Health Sciences, Stockholm University, Stockholm, Sweden; Clinical Epidemiology and Biostatistics, Faculty of Medicine and Health, School of Medical Sciences, Örebro University, Örebro, Sweden; Department of Public Health Sciences, Stockholm University, Stockholm, Sweden; Centre for Health Equity Studies (CHESS), Stockholm University/Karolinska Institutet, Stockholm, Sweden; Aging Research Center (ARC), Karolinska Institutet, Stockholm, Sweden

**Keywords:** sibling bereavement, anniversary reaction, mortality, grief, case-crossover study, Swedish National Registers

## Abstract

Death anniversaries may trigger stress responses that negatively affect health in bereaved individuals. Little is known about such reactions after adult sibling loss. This study examined whether mortality risk increases around the anniversary of a sibling's death. Using Swedish national register data (1990-2016), we conducted a time-stratified case-crossover study including 12 789 adults who experienced sibling loss and later died. Conditional logistic regression estimated associations between mortality and death anniversaries (including pre-anniversary and post-anniversary periods), adjusting for time-invariant confounders. Analyses were stratified by the bereaved's sex and age, the sibling's sex, sibling order, and whether ≥1 parent was alive at the bereaved's death. Among women, mortality risk was lower on the anniversary date (OR, 0.44; 95% CI, 0.21-0.93), and in the period from 1 day before and up to the anniversary date for women who lost a younger or same-age sibling (OR, 0.45; 95% CI, 0.20-1.00). In contrast, men bereaved before age 50 years had a heightened risk in the period ranging from 12 days before and up to the anniversary (OR, 1.40; 95 % CI, 1.05-1.86). Overall, sibling-death anniversaries were not associated with elevated mortality, though observed sex- and age-specific patterns merits further investigation.

## Teaser

Key messages:

- Anniversaries following sibling death might not trigger anniversary reactions to the same extent as child and parental death. Women who experienced sibling death had lower mortality risk around the anniversary.
- Men who experienced sibling death before age 50 years had a heightened risk in the period ranging from 12 days before and up to the anniversary, consistent with an anticipatory anniversary reaction.
- Sibling bereavement can still affect health, but risks may emerge outside the immediate anniversary window and differ by sex and age.

## Introduction

Research shows that the death of a family member can be damaging for the physical and mental health of bereaved individuals.^1^^-^^6^ These health effects may arise from acute psycho-physiological stress and long-term changes in health behaviors, such as smoking and increased alcohol consumption, as well as poor diet and lack of exercise.^7^ Additionally, grief influences emotional states, potentially leading to psychological and psychosomatic health issues, depression, and suicide.^7^^-^^11^

Siblings frequently share one of the longest and most profound relationships in life.^4^^,^^5^^,^^12^^,^^13^

Sibling loss across the life course is linked to various adverse health outcomes, including complicated grief, behavioral and emotional problems, depression, psychosomatic symptoms, early cardiovascular disease, increased risk of suicide, stroke, ischemic heart disease, late-life depression, and cognitive decline among bereaved individuals.^4^^,^^5^^,^^11^^,^^14^^-^^17^

Beyond typical post-loss trajectories, anniversary reactions have been documented. Symbolic dates may trigger stress responses that in turn contribute to acute health deterioration, characterized by spikes in psychological, somatic, and behavioral problems around the anniversary date.^3^^,^^7^^,^^18^^,^^19^ Previous studies have reported increased substance-use disorder and suicide-related behavior close to anniversaries of parental or child deaths.^3^^,^^7^^,^^8^^,^^20^ Such temporary peaks, independent of shared genetics or environment, may signal a causal effect of grief.^7^

As research on anniversary reactions following sibling death is limited, we examined all-cause mortality in adult siblings around the anniversary of a sibling’s death using a time-stratified case-crossover design that controls for time-invariant confounding by using each participant as their own control. We further explored whether any patterns varied by the deceased sibling’s sex (i.e., sister or brother), the age of the bereaved at loss (younger than 50 years; age 50 years or older), sibling birth order (death of older vs younger sibling), and whether parents were still alive (≥1 living parent vs none).

## Methods

### Study population

Our data consist of all individuals aged 16 years and older residing in Sweden at any time between 1990 and 2016, recorded in the Longitudinal Integrated Database for Health Insurance and Labour Market Studies (LISA) (*n* = 13 030 439). For this study, the data were restricted to persons aged ≥16, and individuals younger than 16 years were therefore not included in our dataset. We first excluded 5 780 685 individuals for whom mother and father could not be identified. These were individuals who had lost at least one parent before 1990, individuals born abroad, and those born before 1932. Then, we clustered individuals having the same mother and father, for a total of 3 632 533 clusters. Individuals who were only children were removed, leaving a total of 2 449 510 sibling clusters. We identified 126 432 clusters where at least one sibling died and, for each sibling group, we identified the sibling who died first and defined the date of their death as the anniversary date for the other siblings. To avoid overlapping exposures and dependence between anniversaries, we assigned each bereaved individual a single index bereavement, defined as the first sibling death observed in their family during follow-up; all anniversaries were anchored to this date. Subsequent sibling deaths, if any, were not analyzed. A total of 245 144 bereaved siblings were assigned an anniversary date. Further, we removed 11 244 individuals experiencing sibling death before the age of 16, and 30 individuals who died on the same day as the sibling. Finally, we selected the bereaved siblings who subsequently died, with deaths occurring before December 31, 2016. Thus, our final analytical population included 12 789 individuals who experienced the death of a sibling during adulthood and subsequently died. Figure S1 displays a flowchart of the study population.

The study was approved by the Swedish Ethical Review Authority (decision 2019-06175 and decision 2024-03676-01).

### Study design

A time-stratified case-crossover design was implemented.^21^^,^^22^ For each bereaved sibling in the data, we defined their death day as the case day. We then defined control days as the same weekdays within the same month in which the death occurred. For example, if an individual died on Friday, January 24, 2020, this day is set to be the case day. Control days are set to the other Fridays of the same month of the same year, that is, January 3, 10, 17, and 31, 2020. Thus, the number of control days for each case day can vary between 3 and 4. The design controls for time-invariant confounders by having each individual act as their own control. This approach ensures case and control days are aligned by weekday and short-term seasonal patterns, thus controlling for time-varying confounding. This within-individual comparison strengthens causal inference by reducing confounding and allowing us to isolate the timing-specific effect of anniversaries on mortality.

### Exposures

We created a series of 29 dummy exposure variables relative to the index bereavement date to investigate how the risk of death was associated with the anniversary of a sibling’s death, including 14 days preceding the anniversary, and 14 days following it.^22^ For example, to examine the risk of all-cause mortality on the anniversary day, a dummy exposure variable was created to have value 1 if the case (or the control) day coincided with the anniversary, 0 otherwise. To examine the risk over the period of two days including the day before the anniversary day and the anniversary day, the second dummy exposure variable was assigned a value 1 if the case (or the control) day fell in this period, 0 otherwise. The number of days was increased up to 14 days before the anniversary, and analogous variables were created for periods up to 14 postanniversary days.

### Statistical analysis

We implemented conditional logistic regression models and estimated odds ratios (ORs) with 95% CIs to quantify the associations between the anniversary of sibling death (or pre-anniversary/post-anniversary periods) and the risk of death among bereaved siblings. The models were implemented separately for each of the 29 exposure variables. The OR can be interpreted as the ratio of the odds of death when an individual is exposed (to the anniversary of sibling death or to a pre-anniversary/post-anniversary period) and the odds of death when the individual is not exposed. Standard errors were adjusted for the clustered structure of the case-crossover data.

All analyses were stratified by the sex of the bereaved sibling. Moreover, we ran analyses stratified by the following potential effect modifiers: sex of the deceased sibling, the bereaved sibling’s age at loss (younger than 50 years; 50 years or older), by losing an older or younger sibling, and by parent(s) being alive or not at the time of the bereaved’s death (≥1 living parent vs none). To assess robustness, we reestimated the sex stratified models spacing control days 14 and 28 days apart from each other. In this way, we avoided dependance between case’s and control’s periods (for periods longer than 7 days) and we avoided selecting reference days that might be affected by anticipatory or post-anniversary effects. Further, we repeated analyses by restricting to deaths occurring within 5 years of the sibling’s death to investigate whether associations are stronger closer to bereavement. Finally, we applied a Bonferroni adjustment across day-specific estimates to account for multiple testing. Two-sided *P* values < .05 were considered statistically significant. All analyses were performed using Stata 15.1 (StataCorp).

## Results

Table 1 summarizes the characteristics of the 12 789 individuals who experienced the death of a sibling in adulthood and subsequently died, of whom 7764 (61%) were men. Median age at the time of death was 65 years (IQR, 58-71 years) and median time since the sibling’s death and their own death was 6 years (IQR, 3-12 years). Overall, 60.5% lost a brother and 73.6% experienced sibling death after the age of 50, while 50.0% lost a younger/same-age sibling. At the time of their own death, 69.2% had no living parent. It was more common to be ever married (73.2%), and to have secondary education (43.2%).

**Table 1:** Characteristics of individuals who lost a sibling during adulthood and who subsequently died (*n* = 12 789), Sweden, 1990-2016.

	**Women (*n* = 5025)**	**Men (*n* = 7764)**	**Total (*N*= 12 789)**
Age at death, median (IQR), years	66 (59-72)	65 (58-71)	65 (58-71)
Time since loss, median (IQR), years	6 (3-12)	6 (3-12)	6 (3-12)
	***n*, %^a^**	***n*, %^a^**	***n*, %^a^**
Relative age to deceased sibling			
Younger or same age	2524 (50.2)	3875 (49.9)	6399 (50.0)
Older	2501 (49.8)	3889 (50.1)	6390 (50.0)
Age at sibling death			
Before age 50 years	1204 (24.0)	2175 (28.0)	3379 (26.4)
Age 50 years or older	3821 (76.0)	5589 (72.0)	9410 (73.6)
Sibling’s sex			
Sister	2123 (42.2)	2935 (37.8)	5058 (39.5)
Brother	2902 (57.8)	4829 (62.2)	7731 (60.5)
Number of living parents at the time of the bereaved’s death			
≥1 living parent	3531 (70.3)	5321 (68.5)	8852 (69.2)
No living parent	1494 (29.7)	2443 (31.5)	3937 (30.8)
Civil status			
Never married	1015 (20.2)	2397 (30.9)	3412 (26.7)
Ever married	4006 (79.7)	5356 (69.0)	9362 (73.2)
Missing/unknown	4 (0.1)	11 (0.1)	15 (0.1)
Education			
Primary	1951 (38.8)	3322 (42.8)	5273 (41.2)
Secondary	2214 (44.1)	3317 (42.7)	5531 (43.2)
Tertiary	799 (15.9)	1043 (13.4)	1842 (14.4)
Missing/unknown	61 (1.2)	82 (1.1)	143 (1.1)

a Percentages are column percentages within sex; totals show the overall distribution.

**Figure 1:**
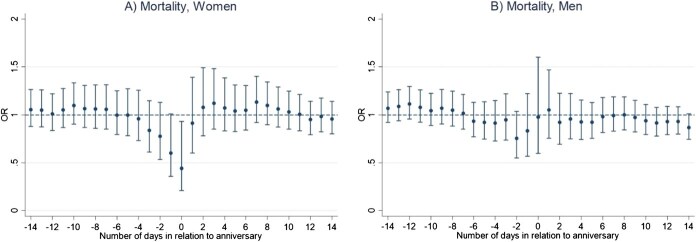
Mortality risk in relation to the anniversary of a sibling’s death, by sex of bereaved individual, Sweden 1990-2016, Sweden 1990-2016, estimated using conditional logistic regression in a time-stratified case-crossover design. (A) Day 0 represents the anniversary of the sibling’s death. (B) Estimates are calculated for the anniversary and for each of the 14 days before and 14 days after the anniversary.

**Figure 2:**
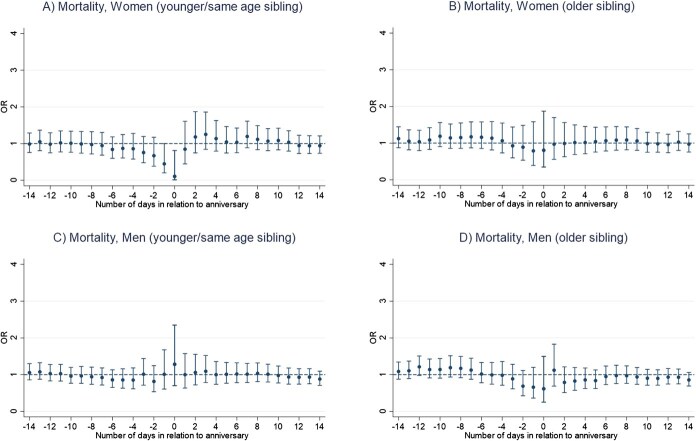
Mortality risk around the anniversary of a sibling’s death, by age relation to the deceased sibling and sex of the bereaved individual, Sweden 1990-2016, estimated using conditional logistic regression in a time-stratified case-crossover design. (A) Day 0 represents the anniversary of the sibling's death. (B) Estimates are calculated for the anniversary and for each of the 14 days before and 14 days after the anniversary.

**Figure 3:**
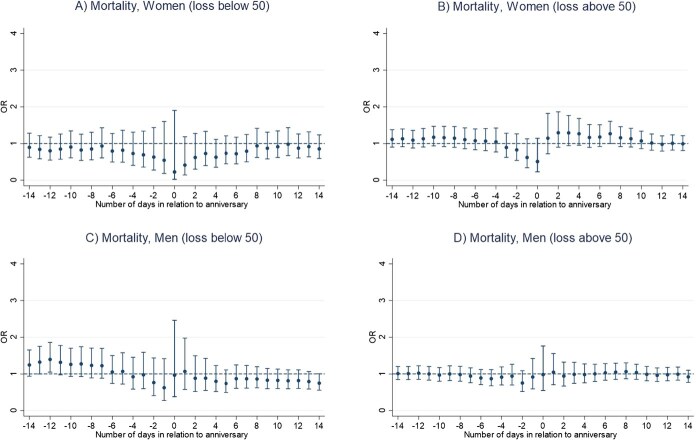
Mortality risk around the anniversary of a sibling’s death, by bereaved individual’s age at loss (younger than 50 years; 50 years or older) and sex, Sweden 1990-2016, estimated using conditional logistic regression in a time-stratified case-crossover design. (A) Day 0 represents the anniversary of the sibling’s death. (B) Estimates are calculated for the anniversary and for each of the 14 days before and 14 days after the anniversary.

**Figure 4:**
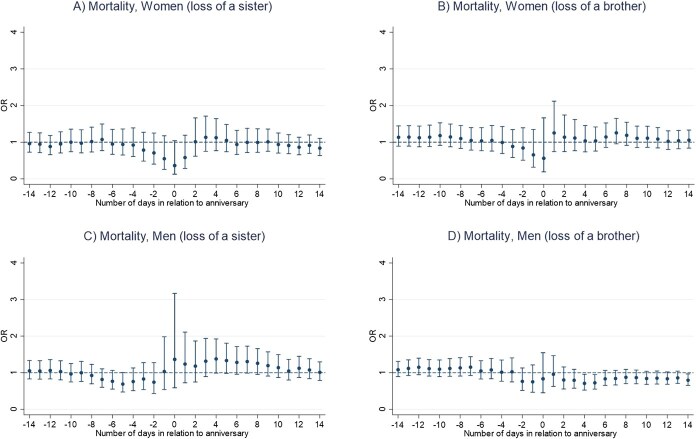
Mortality risk around the anniversary of a sibling’s death, by sex of the deceased sibling and the bereaved individual, Sweden 1990-2016, estimated using conditional logistic regression in a time-stratified case-crossover design. (A) Day 0 represents the anniversary of the sibling’s death. (B) Estimates are calculated for the anniversary and for each of the 14 days before and 14 days after the anniversary.

**Figure 5:**
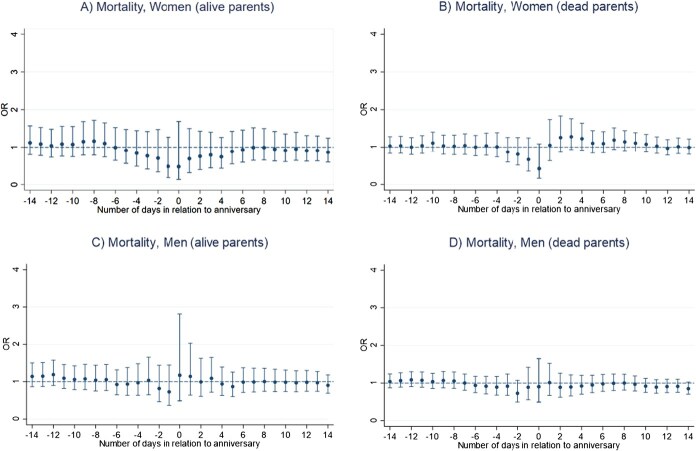
Mortality risk around the anniversary of a sibling’s death, by parent(s) being alive or not, Sweden 1990-2016, estimated using conditional logistic regression in a time-stratified case-crossover design. (A) Day 0 represents the anniversary of the sibling’s death. (B) Estimates are calculated for the anniversary and for each of the 14 days before and 14 days after the anniversary.

Results of the main conditional logistic regression models indicate that the anniversary of a sibling’s death is not associated with an increased mortality risk in bereaved siblings, for neither men nor women (Figure 1A and B). We rather found a lower mortality risk on the anniversary date for women (OR, 0.44; 95% CI, 0.21-0.93) (Figure 1A and B; Tables S1 and S2). Number of deaths for each analysis ranged from 95 to 796 among men and from 68 to 541 among women (Tables S1 and S2). Moreover, we found a lower mortality risk in the period from one day before and up to the anniversary date for women who lost a younger or same age sibling (OR, 0.45; 95% CI, 0.20-1.00) (Figure 2A). In contrast, we observed a significant increase in mortality risk among men who were bereaved before age 50, in the period ranging from 12 days before and up to the death anniversary (OR, 1.40; 95% CI, 1.05-1.86) (Figure 3C). No other statistically significant associations were found in analyses stratified by sibling order (Figure 2A-D), age at loss (Figure 3A-D), the deceased sibling's sex (Figure 4A-D), or by parent(s) being alive or not at the time of the bereaved's death (Figure 5A-D).

### Sensitivity analyses

In the reestimated sex-stratified models with control days spaced 14 and 28 days apart from each other (Figures S2 and S3), patterns of associations were similar to the main analysis but the reduced mortality in women on the anniversary was no longer statistically significant. Limiting follow-up to ≤5 years since loss increased the magnitude of the anniversary reduction among women, but widened confidence intervals and rendered the association nonsignificant (Figure S4). Applying a Bonferroni adjustment to address multiple testing likewise led to nonsignificant associations (Figure S5).

## Discussion

We found no increased mortality risk during the anniversary following sibling death, implying that such dates might not trigger grief reactions to the same extent as anniversaries following the death of a parent or child.^3^^,^^7^^,^^20^ Instead, we observed a lower mortality risk on the anniversary date for women, as well as in the period including one day prior to and the anniversary date for women who lost a younger or same age sibling. In contrast, men bereaved before age 50 years showed an elevated mortality risk in the period ranging from 12 days before and up to the anniversary. Sensitivity analyses using control days spaced further apart only partially confirmed the risk reduction on the anniversary among women, suggesting that our findings may reflect short-term fluctuations rather than broader temporal trends. This supports the interpretation that the observed associations can be interpreted as psychological responses locally triggered by the anniversary.

The lower mortality risk observed in women around a sibling's death anniversary is a novel finding that warrants further exploration. Interestingly, this pattern contrasts with evidence from other bereavement contexts, where studies have found heightened anniversary risks among women bereaved of a parent or child.^3^^,^^7^^,^^20^ A plausible explanation lies in family dynamics and gender-specific sibling roles: women, particularly older sisters, commonly take on key caregiving and supportive roles within the family.^23^^,^^24^ When parents are still alive, the prospect of a second child dying on the same date could be especially distressing. Surviving daughters may, driven by a heightened sense of purpose and responsibility to family, consciously or not, modify behaviors or seek care in ways that defer short-term risk around that day.^25^^,^^26^ In this sense, female siblings may adopt short-term protective behaviors around the anniversary leading to the lower risk observed in women. We acknowledge that these interpretations are speculative and, because results in our sensitivity analyses were not statistically significant, the possibility of chance findings cannot be excluded. Therefore, we encourage future studies with richer family-context data to further investigate how gendered caregiving and family dynamics, including gender specific sibling roles, might be associated with the timing of anniversary reactions.

The heightened risk in the period ranging from 12 days before and up to the anniversary among younger bereaved men might indicate anticipatory anniversary reactions, whereby distress and health risks increase in the lead-up to an anniversary date rather than on the date itself.^27^ A Swedish register study found higher substance-use disorder and suicide-related behaviors in the month before a parent’s death anniversary, particularly in males, and a recent systematic review concluded that anticipatory elevations in psychopathology and mortality can begin days or weeks ahead of significant dates.^3^^,^^28^

To our knowledge, no other studies have examined the anniversary reaction in relation to adult sibling bereavement and its association with mortality risk. Consistent with our findings, a population-based Danish study on individuals bereaved by suicide similarly found no overall heightened risk of suicidal behavior during emotionally significant anniversaries, such as the date of death or the deceased person's birthday.^29^ Another study, however, found that adolescents and young adults who experienced parental loss were at an increased risk of substance use disorders and suicide-related behaviors, with women and girls being particularly affected.^3^ Further, a study of parental mortality around significant dates found that mothers experienced a heightened mortality risk during the week of their child's death anniversary, whereas fathers showed a reduced mortality risk around both birth and death anniversaries.^7^ Our findings align with these observations in men, as they suggest no increased mortality risk around anniversaries. However, our results suggest that women may also not experience an elevated mortality risk on and around anniversaries following a sibling's death and may even have a lower risk. As these findings challenge prior assumptions about heightened vulnerability in women, further research is needed to better understand gender differences in bereavement-related outcomes in the context of sibling loss.

### Strengths and limitations

To the best of our knowledge, this is the first large-scale study using data from national registers to investigate whether mortality increases among adult siblings around the anniversary of a sibling’s death. The time-stratified case-crossover design controls for all time-invariant confounding by using each participant as their own control. This design also allowed us to account for potential seasonal effects. Additionally, the study benefits from minimal loss to follow-up, and the comprehensive Swedish national registers provide highly accurate and complete data on both mortality and the timing of death. This approach strengthens the validity of our findings and reduces the risk of confounding, offering a robust analysis of the association between sibling death anniversaries and mortality.

Some limitations should be noted. As our study includes only siblings who experienced the death of a sibling and subsequently died themselves, our dataset is inherently limited. In turn, this reduces precision, yielding wide confidence intervals and limiting power to detect small associations. As a result, there is a possibility that the findings could be influenced by chance, particularly in the smaller subgroups. Consequently, these results should be interpreted with caution and further research is needed to confirm the robustness of the observed associations.

Further, due to data limitations, we did not investigate anniversary reactions around other significant dates, such as holidays, or birthdays of the deceased sibling.^7^ Another consideration is that we did not examine cause-specific patterns due to limited events in the day-specific analyses. The impact of bereavement may differ depending on whether the loss was sudden and unexpected (e.g., suicide or accidents), or occurred after a prolonged illness (e.g., cancer), where family members may have had more time to adjust and prepare. Investigating all-cause mortality around anniversaries among bereaved siblings means we may have missed relevant associations with specific causes of death, such as suicide, stroke, or ischemic heart disease, which previous studies have linked to adult sibling bereavement.^5^^,^^16^ Future research could benefit from incorporating cause-specific mortality data to explore whether anniversary reactions are more pronounced following certain types of sibling loss.

Moreover, the registers contain no information on relationship quality (e.g., perceived closeness or frequency of contact) or household composition. Although these factors are unlikely to confound our within-person design, they may modify anniversary effects; for example, stronger reactions may occur among siblings with closer bonds. Because sibling relationships range from little to no contact to very close, increases and decreases in short-term risk may offset one another in population averages. Future work combining register data with survey or interview measures of relationship dynamics could test such heterogeneity.

In our main analysis, we found that mortality did not increase around the anniversary of a sibling’s death. Rather, we observed a lower mortality risk in women on the anniversary date, and in the period from one day before and up to the anniversary date for women who lost a younger or same-age sibling. In contrast, men bereaved before age 50 years exhibited increased mortality risk in the period ranging from 12 days before and up to the anniversary, possibly indicating an anticipatory reaction. These interesting sex- and age-specific patterns may reflect differences in family roles, coping strategies, or emotional closeness that warrant further investigation. Future research should look beyond mortality, examining health-care utilization, sick leave, health behaviors, mental-health diagnoses, and hospitalizations before, on, and after anniversaries, to include sibling bereavement in all ages, and to deepen understanding of consequences of sibling loss.

## Supplementary Material

Web_Material_kwaf213

## Data Availability

Access to the data requires ethical approval from the Swedish Ethical Review Authority and permission from the relevant authority due to the sensitive nature of individual-level health data. Data may be available from the authors upon reasonable request and with appropriate approvals.
