# Supplementary material for: All-cause mortality around the anniversary of a sibling’s death: findings from Swedish National Register Data
Source: Am J Epidemiol. 2025 Sep 30;195(3):824–31. doi: 10.1093/aje/kwaf213 (PMC13017226; doi:10.1093/aje/kwaf213)
Supplement: Web_Material_kwaf213 [file web_material_kwaf213.docx]

**All-cause Mortality Around the Anniversary of a Sibling’s Death: Findings from Swedish National Register Data**

Rogne, S., Grotta, A., Liu, C., Berg, L. Saarela, J., Kawachi, I., Hiyoshi, A., Rostila, M.

List of included materials: Table S1 and S2, Figure S1-S5

**Table S1**. Odds ratios (ORs) and 95% confidence intervals (CIs) for mortality on the anniversary of a sibling’s death and for pre-/post-anniversary periods within 14 days before and after the anniversary among bereaved men (n = 7,764), Sweden, 1990–2016.

| **Number of days from anniversary** | **OR (95% CI)** | **Number of deaths** |
| --- | --- | --- |
| -14 | 1.07 (0.92, 1.24) | 779 |
| -13 | 1.09 (0.94, 1.27) | 763 |
| -12 | 1.12 (0.96, 1.30) | 739 |
| -11 | 1.08 (0.92, 1.26) | 728 |
| -10 | 1.05 (0.89, 1.23) | 711 |
| -9 | 1.07 (0.91, 1.27) | 686 |
| -8 | 1.05 (0.88, 1.25) | 667 |
| -7 | 1.02 (0.85, 1.22) | 648 |
| -6 | 0.94 (0.77, 1.13) | 625 |
| -5 | 0.92 (0.75, 1.14) | 524 |
| -4 | 0.92 (0.73, 1.15) | 441 |
| -3 | 0.95 (0.74, 1.22) | 355 |
| -2 | 0.76 (0.55, 1.04) | 260 |
| -1 | 0.83 (0.57, 1.22) | 169 |
| 0 | 0.98 (0.60, 1.60) | 95 |
| 1 | 1.05 (0.76, 1.47) | 200 |
| 2 | 0.92 (0.69, 1.23) | 287 |
| 3 | 0.96 (0.75, 1.23) | 379 |
| 4 | 0.93 (0.74, 1.16) | 473 |
| 5 | 0.92 (0.76, 1.13) | 572 |
| 6 | 0.98 (0.82, 1.18) | 649 |
| 7 | 0.99 (0.83, 1.19) | 663 |
| 8 | 1.00 (0.84, 1.19) | 684 |
| 9 | 0.97 (0.82, 1.15) | 705 |
| 10 | 0.94 (0.80, 1.11) | 718 |
| 11 | 0.92 (0.78, 1.08) | 731 |
| 12 | 0.93 (0.80, 1.09) | 752 |
| 13 | 0.93 (0.80, 1.09) | 772 |
| 14 | 0.87 (0.75, 1.01) | 796 |

**Table S2**. Odds ratios (ORs) and 95% confidence intervals (CIs) for mortality on the anniversary of a sibling’s death and for pre-/post-anniversary periods within 14 days before and after the anniversary among bereaved women (n = 5,025), Sweden, 1990–2016.

| **Number of days from anniversary** | **OR (95% CI)** | **Number of deaths** |
| --- | --- | --- |
| -14 | 1.06 (0.88, 1.27) | 532 |
| -13 | 1.05 (0.88, 1.26) | 522 |
| -12 | 1.01 (0.84, 1.22) | 500 |
| -11 | 1.05 (0.87, 1.28) | 489 |
| -10 | 1.10 (0.90, 1.34) | 481 |
| -9 | 1.07 (0.87, 1.31) | 463 |
| -8 | 1.06 (0.86, 1.32) | 455 |
| -7 | 1.06 (0.85, 1.32) | 443 |
| -6 | 1.00 (0.80, 1.25) | 433 |
| -5 | 1.00 (0.78, 1.28) | 376 |
| -4 | 0.96 (0.73, 1.26) | 310 |
| -3 | 0.84 (0.61, 1.15) | 250 |
| -2 | 0.78 (0.53, 1.13) | 184 |
| -1 | 0.60 (0.36, 1.01) | 112 |
| 0 | 0.44 (0.21, 0.93) | 68 |
| 1 | 0.91 (0.60, 1.39) | 130 |
| 2 | 1.08 (0.78, 1.49) | 201 |
| 3 | 1.12 (0.85, 1.48) | 267 |
| 4 | 1.07 (0.83, 1.38) | 327 |
| 5 | 1.04 (0.83, 1.31) | 396 |
| 6 | 1.05 (0.84, 1.31) | 437 |
| 7 | 1.14 (0.92, 1.40) | 451 |
| 8 | 1.10 (0.90, 1.35) | 465 |
| 9 | 1.06 (0.87, 1.29) | 476 |
| 10 | 1.03 (0.85, 1.25) | 485 |
| 11 | 1.01 (0.84, 1.22) | 497 |
| 12 | 0.95 (0.79, 1.14) | 514 |
| 13 | 0.99 (0.82, 1.18) | 530 |
| 14 | 0.96 (0.80, 1.14) | 541 |


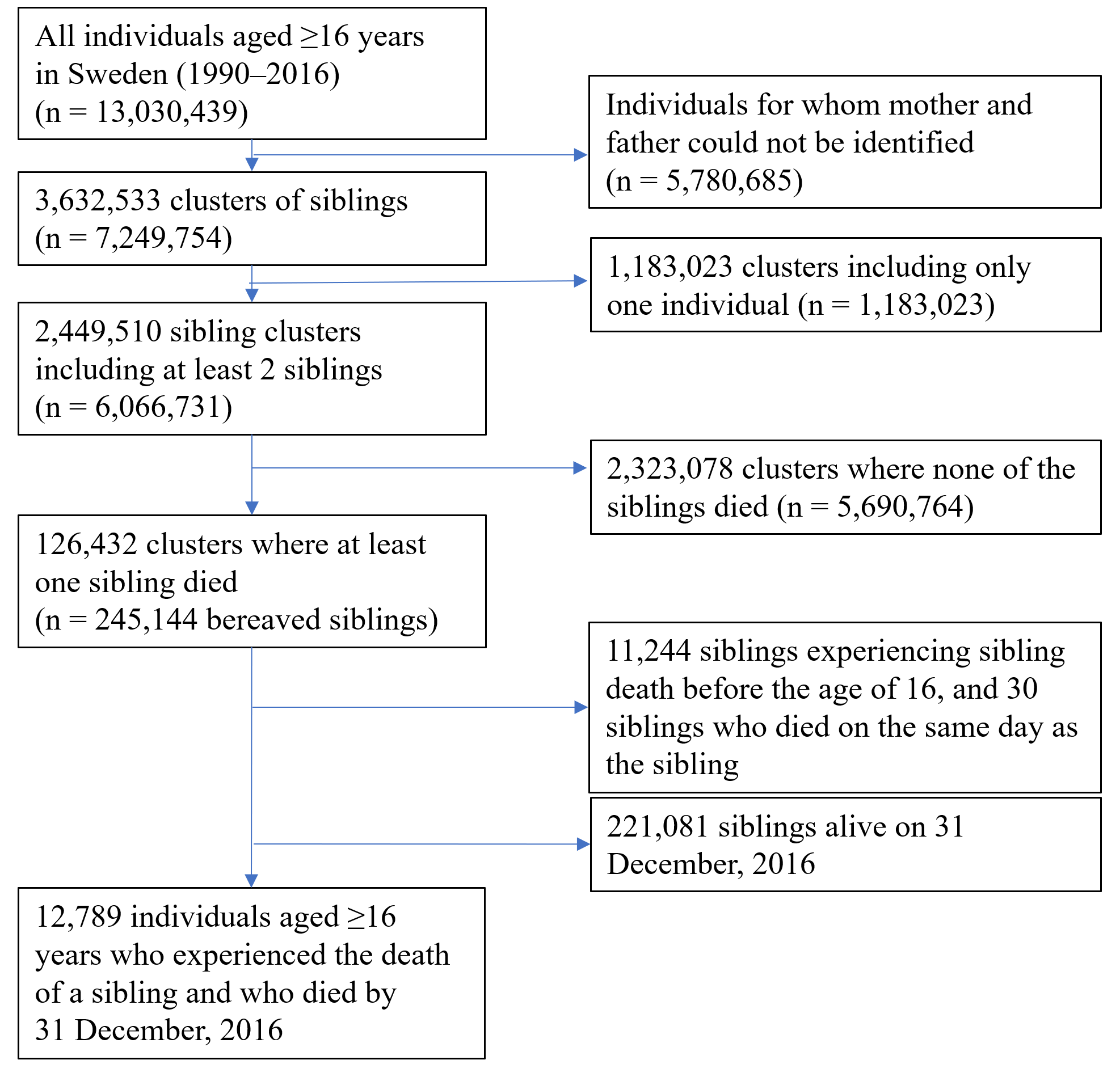


**Figure S1**: Flowchart of study population, Sweden 1990–2016.


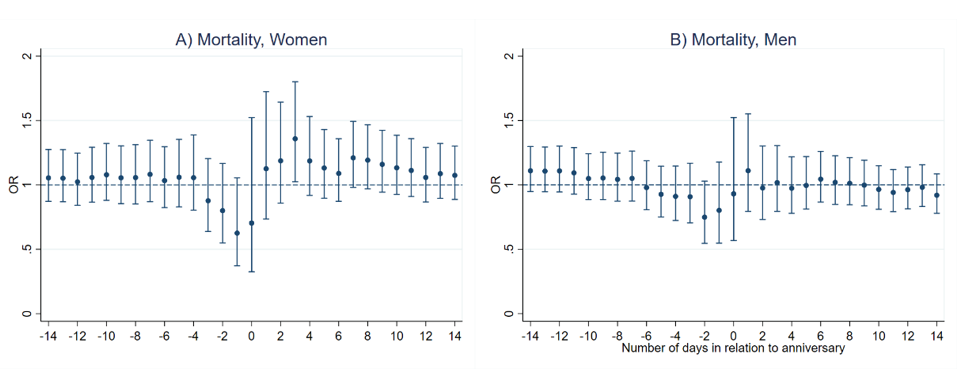


**Figure S2**: Mortality risk in relation to the anniversary of a sibling’s death, by sex of bereaved individual, estimated using conditional logistic regression in a time-stratified case-crossover design, selecting controls 14 days apart from each other.


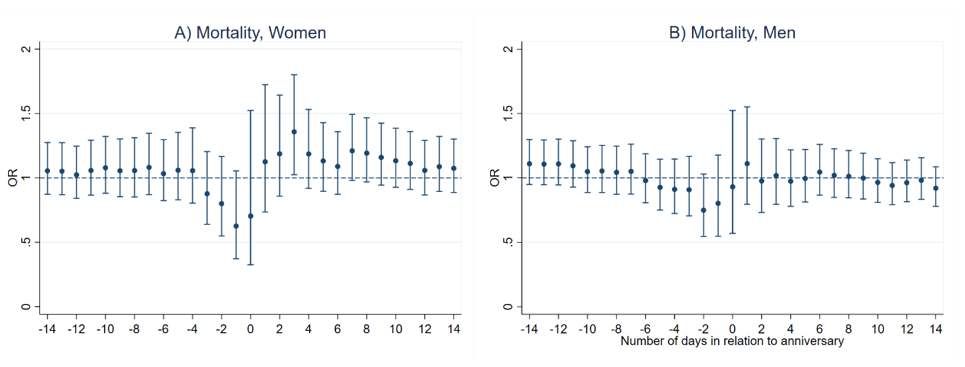


**Figure S3**: Mortality risk in relation to the anniversary of a sibling’s death, by sex of bereaved individual, estimated using conditional logistic regression in a time-stratified case-crossover design, selecting controls 28 days apart from each other.


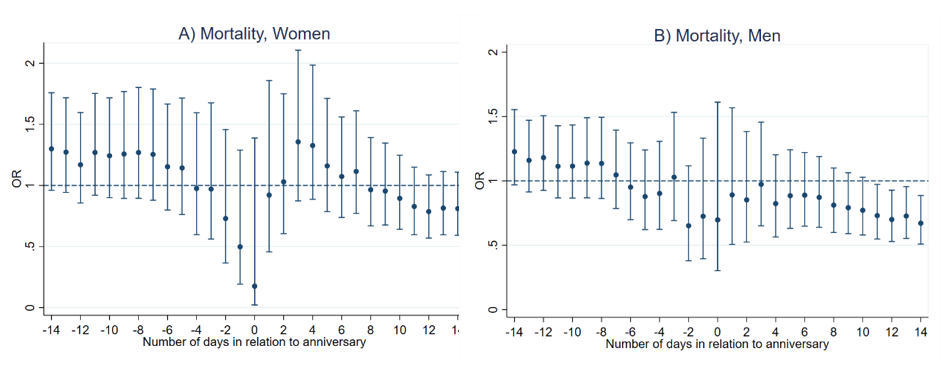


**Figure S4**: Mortality risk in relation to the anniversary of a sibling’s death, by sex of bereaved individual, estimated using conditional logistic regression in a time-stratified case-crossover design, restricting to deaths occurring in the first 5 years following sibling´s death.


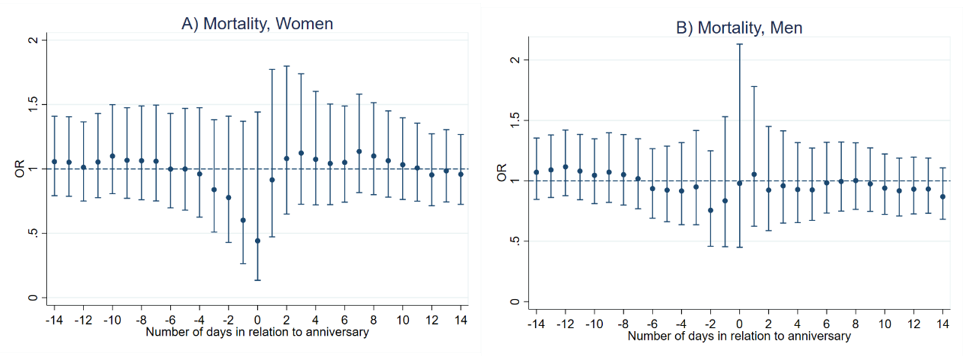


**Figure S5**: Mortality risk in relation to the anniversary of a sibling’s death, by sex of bereaved individual, estimated using conditional logistic regression in a time-stratified case-crossover design, using Bonferroni adjustment for addressing multiple testing.
